# Harnessing Photon Recoil for Enhanced Torque on Light-Driven Metarotors

**DOI:** 10.1021/acs.nanolett.4c06410

**Published:** 2025-03-03

**Authors:** Mahdi Shanei, Gan Wang, Peter Johansson, Giovanni Volpe, Mikael Käll

**Affiliations:** † Department of Physics, Chalmers University of Technology, 412 96 Gothenburg, Sweden; ‡ Department of Physics, University of Gothenburg, 412 96 Gothenburg, Sweden; § School of Science and Technology, Örebro University, 701 82 Örebro, Sweden

**Keywords:** metasurfaces, microrobots, microrotors, optical torque, optical force

## Abstract

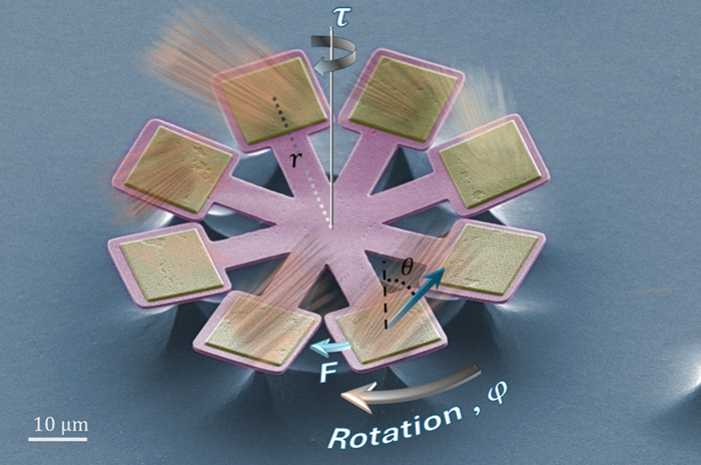

Contact-free rotation of microscopic objects in aqueous environments based on optical forces is a powerful concept in the development of light-driven microrobots, micromachines, torque transducers, and rheological sensors. Here, we demonstrate freely movable quasi-two-dimensional metasurface rotors with lateral dimensions up to 100 μm while still exhibiting controllable and steady rotation when submerged in water. The metarotors utilize photon recoil to produce strong optical torque by deflecting low-intensity laser light toward high angles via long lever arms, which amplify the creation of orbital angular momentum. We find that the torque generated by a single metarotor can be used to rotate hundreds of passive microparticles present in solution, suggesting potential applications as particle mixers in microfluidics and microbiology. Further development might involve utilizing metarotors as components in future microrobots for biomedicine and beyond.

Microrobots and micromotors able to move and perform tasks in complex environments are expected to become important tools in future nanotechnology and biomedicine, potentially providing novel capabilities for minimally invasive surgery, targeted therapy, and cellular manipulation.^1,2^ Consequently, over the past decades, significant research efforts have been devoted to developing methods for efficient microrobot control based on a variety of magnetic,^3^ electric, acoustic,^4^ and optical actuation mechanisms.^5−8^ Optical actuation, based on photothermal effects or optical forces, has significant advantages in terms of spatial and temporal resolution. Additionally, it benefits from widely accessible optomechanical technologies, such as powerful laser sources.^9^ Optical tweezers are arguably the most successful example of optical actuation, allowing exquisite control of microparticles by converting light momentum directly into mechanical movement by utilizing focused laser beams.

Optically driven microrotors constitute a special class of microrobots with potential applications across diverse fields of science,^10^ including sensing,^11^ microfluidics,^12^ and in characterizing the dynamics of complex systems.^13,14^ Two primary methods for rotating microscopic objects using optical forces have been reported in the literature. First, the rotor can be gripped at specific points by movable optical tweezers, causing it to rotate along with the rotating laser traps.^15^ However, this approach requires additional components to steer the optical tweezers, such as spatial light modulators, that add complexity to the system. Second, the light-object system can be constructed such that it is three-dimensionally chiral.^16,17^ This can be achieved by using light that carry spin and/or orbital angular momentum, which provides torque but requires precise control of the polarization and/or spatial phase profile of the incident laser beam.^18−20^ It can also be achieved by designing the microrotor such that it deflects or reflects light azimuthally, thereby effectively converting the incident radiation pressure force to optical torque.^21−26^ The latter approach has the significant advantage that the incident beam can, in principle, be unstructured and unpolarized. However, the advantage comes with the challenge of having to fabricate three-dimensionally “twisted” rotor structures on the micrometer-scale, which has previously primarily been achieved using low-throughput two-photon polymerization lithography techniques.

Here we explore the use of optical metasurfaces, specifically amorphous silicon (a-Si) metagratings, to generate torque through azimuthal beam deflection. Our “metarotor” concept, illustrated in Figure 1a, build on recent innovations that involve the integration of artificial directional scatterers into an optically thin structure.^27−29^ A metarotor is constructed from pairs of metagratings positioned at the periphery of the rotor via long SiO_2_ bars, which act as lever arms for the optical force. Each metagrating *i* is designed and oriented to deflect normally incident plane waves such that a lateral photon recoil force *F*_*i*_ is induced in the azimuthal direction φ. The resulting net torque τ = ∑_*i*_*F*_*i*_*r*_*o*_, where *r*_*o*_ is the length of the lever arms, drives in-plane rotation around the metarotor center of mass. The fabrication method, based on electron-beam lithography, is fully scalable, and offers potential efficiency improvements since the metarotors can be made very thin, which implies low drag in a liquid environment. The compact size, the high deflection angle θ, and the high deflection efficiency of the metagratings, then makes it possible to construct freely movable rotating microrotors. We have recently used this approach to construct small “metaspinners” equipped with a single pair of metagratings and with a total particle diameter of 8 μm,^30^ which is similar to the size of microrotors reported previously.^25,26,29,31^ In contrast, the metarotors demonstrated here contain up to eight metagratings and their diameters can be as large as 100 μm, which implies that the generated optical torque is substantially increased compared to those earlier works. In the following, we investigate the optical, thermal, and hydrodynamic behavior of these large metarotors and demonstrate their ability to collect and rotate hundreds of passive microparticles in solutions.

**Figure 1:**
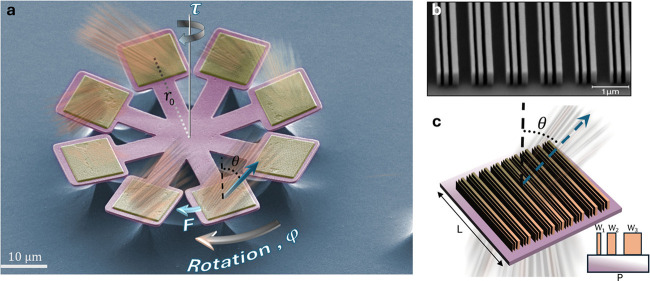
Light-driven metarotors. (a) False color SEM image of a quadruple metarotor ready to be released from the substrate. Four pairs of a-Si metagratings, positioned at the tips of long SiO_2_ bars, deflect normally incident light at high angles θ. Each metagrating is oriented to induce a lateral photon recoil force *F* in the azimuthal direction φ. This results in a net torque with magnitude τ = 8*Fr*_*0*_ due to the lever arms with length *r*_*0*_, which forces the metarotor to rotate in-plane around its center of mass. (b) SEM image of a metagrating and (c) schematic illustration of its geometry. A metagrating has periodicity *P* = 817 nm, and each unit cell contains three 490 nm tall a-Si ridges with widths *W*_1_ = 90 nm, *W*_2_ = 130 nm, *W*_3_ = 190 nm, and separation 100 nm. Each metagrating is supported by a square SiO_2_ substrate with typical side-length *L* = 10 μm. The design wavelength is λ_0_ = 1064 nm.

We fabricated metarotors using a combination of electron beam lithography and etching (see Methods and Figures S1 and S2 in the Supporting Information). We focused on the structure type illustrated in Figure 1, where the gaps between the long SiO_2_ bars allows the etchant to easily reach underneath all parts of the structure, thus facilitating release into solution. Variants with similar benefits, for example ring-shapes (Figure S2j), are possible but were not investigated in detail. We used the metagrating design recently reported in ref^30^ to generate torque. The metagrating unit cell consists of three parallel a-Si (*n* = 3.8) ridges with different widths but fixed height and gap size (see Figure 1c and Methods). At the design wavelength, λ_0_ = 1064 nm, the subwavelength periodicity, *P* = 817 nm, gives first-order diffraction at θ = 64° in SiO_2_ and θ = 70° after refraction at the SiO_2_/water interface.

The maximum orbital optical torque that can be generated by a pair of metagratings oriented to deflect normally incident plane waves in opposite lateral directions is approximately given by τ = 2*r*_0_*F*_0_, corresponding to the case of 100% deflection at θ = 90°. Here *r*_0_ is the length of the lever arm, measured from the metarotor center of mass to the center of each metagrating, and *F*_0_ is the radiation pressure force that the incident field would generate on a completely absorptive object with the same geometrical cross section as the metasurface area. In reality, θ < 90° and the metasurfaces are only able to deflect a polarization dependent fraction of the incident light momentum in the preferred direction. In the following, we focus on linearly polarized incidence, for which torque generation is dominated by orbital angular momentum transfer from the diffracted waves. We can then express the torque as τ = 2*r*_0_*F*_0_*f* (φ), where *f* (φ) is a force fraction that depends on the azimuthal angle φ between the metagrating diffraction plane and the plane of polarization or, equivalently, on the azimuthal rotation angle. A simplified analysis leads to *f* (φ) = *f*_*p*_ cos^2^φ + *f*_*s*_ sin^2^φ, where *f*_*p,s*_ = (*T*_*p,s*_^+1^ - *T*_*p,s*_^–1^+*R*_*p,s*_^+1^ - *R*_*p,s*_^–1^)sin(θ) and the *T* and *R* factors are power diffraction efficiencies for transmission and reflection, respectively.^30^ Here, the superscript refers to diffraction order and the subscript refers to polarization within (*p*) or perpendicular to (*s*) the plane of diffraction.

In Figure 2a, we plot *f* (φ) for the fabricated metagrating structure based on finite element simulations of diffraction efficiencies (Figure S3). The simulation was performed for an a-Si metagrating encapsulated by a 1 μm thick SiO_2_ slab surrounded by water and thus includes refraction at the SiO_2_/water interface as well as multiple reflection effects. The force fraction reaches ∼0.6 at *φ =* 0 deg. (*p*-polarization) but decreases to ∼0.25 at 90 deg. (*s*-polarization), which reflects the fact the original design was optimized to maximize *T*_*p*_^+1^ sin(θ).^30^ As a result, the torque generated by a rotor equipped with only two metagratings, connected by a single SiO_2_ bar, will fluctuate as φ varies during rotation. However, more bars can be symmetrically added to the structure to counter this effect. The resulting net torque, τ (φ, *N*) = 2*r*_0_*F*_0_ ∑_*i*=0_^*N*–1^*f* (φ + *iN*/180) for an *N*-bar metarotor, is angle independent for *N* > 1 and simply equals *N* times the average single bar torque, that is .

**Figure 2:**
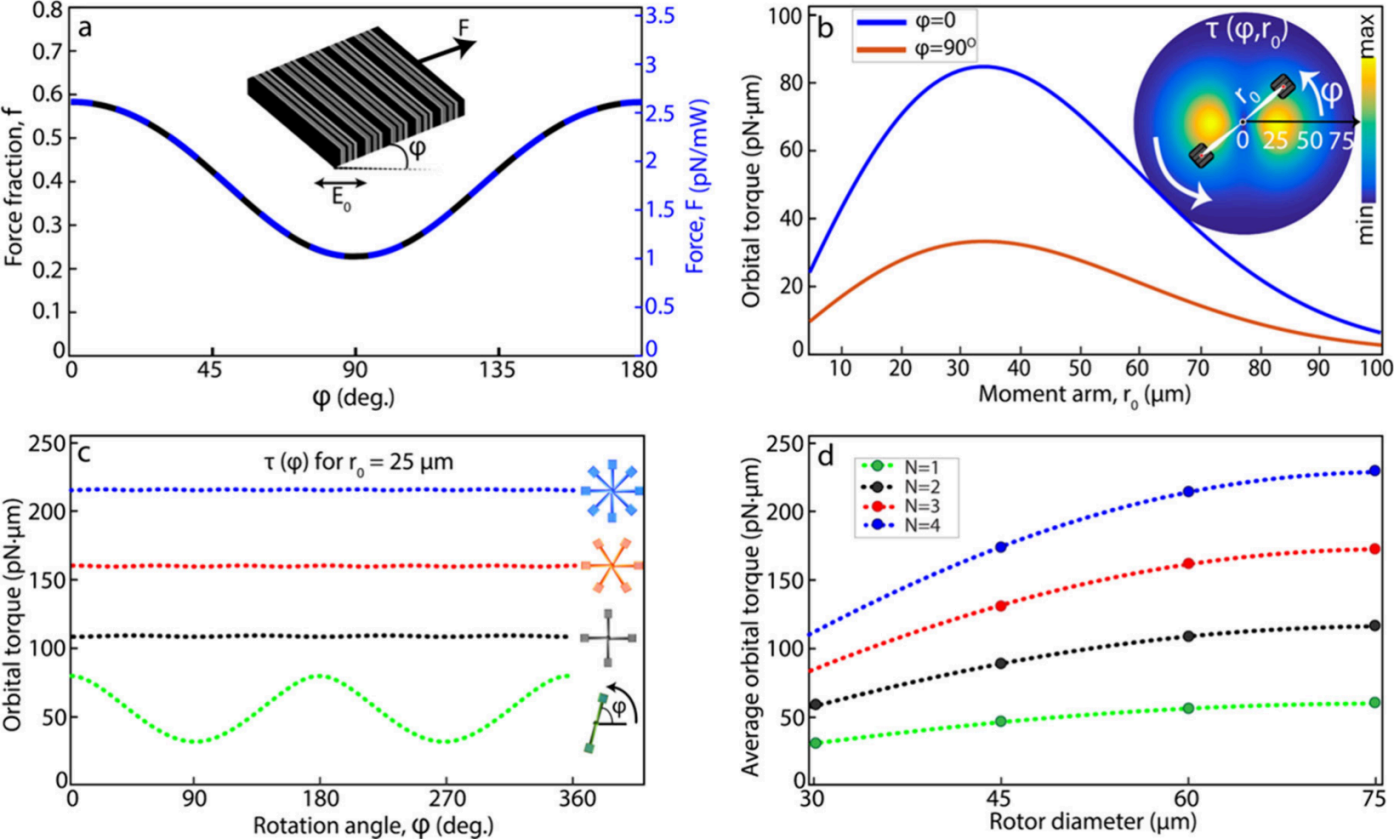
Simulated forces and torques. (a) Metagrating force factor *f* (φ), where φ is the angle between the diffraction and polarization planes, obtained from calculated diffraction efficiencies for a normal incidence and linearly polarized plane wave with wavelength *λ*_*0*_ = 1064 nm. The rightmost *y*-axis indicates the corresponding in-plane recoil force per mW of incident power. (b) Calculated orbital torque τ versus lever arm length *r*_0_ for a single-bar metarotor in a Gaussian beam with beam-waist *w*_0_ = 67.5 μm and peak intensity *I*_0_ = 12 μW/μm^2^. The inset illustrates τ(φ, *r*_0_). (c) τ(*φ)* for *r*_0_ = 25 μm and (d) average torque τ̅ versus rotor diameter for metarotors with *N* = 1, 2, 3, and 4 bars for the same beam parameters as in (b). The rotors in (b–d) are equipped with square metagratings containing 10 diffractive unit cells, and the dots in (d) indicate fabricated samples.

The metarotor concept described above assumes unstructured plane wave illumination. However, any experimental realization will involve a laser field with a laterally varying intensity distribution. Here we focus on the special case of a radially symmetric Gaussian distribution *I* (*r*) = *I*_0_ exp(−2*r*^2^ /*w*_0_^2^), where *I*_0_ is the peak intensity and *w*_0_ is the beam-waist radius. Figure 2b shows the calculated torque versus lever arm length *r*_0_ for a single-bar rotor equipped with square metagratings containing *S* = 10 unit cells. The incident beam parameters are *I*_0_ = 12 μW/μm^2^ and *w*_0_ = 67.5 μm, as in the experiments described in the next section. As expected, the torque first increases with *r*_0_ but then decreases as the metagratings become exposed to lower intensity as they get further away from the focal point, resulting in an optimum near *r*_0_ = *w*_0_/2. We fabricated metarotors equipped with metagratings with *S* = 5, 10, and 15 unit cells, *N* = 1–4 bars, and lever arm lengths *r*_0_ = 10, 17.5, 25, and 32.5 μm. Figure 2c and d summarizes the expected torque generation as a function of angle φ and rotor diameter *D* = 2*r*_0_ + *S·P* for *S* = 10.

Experiments were performed with metarotors immersed in deionized water contained in a thin liquid cell mounted on an inverted microscope and with the λ_0_ = 1064 nm driving laser beam entering from above (see Methods and Figure S4). Figure 3a (Videos 1, 2, 3, 4) shows examples of rotating 60 μm diameter (*r*_0_= 25 μm) metarotors with *N* = 1, 2, 3, and 4 bars and *S* = 10 metagratings oriented to generate counterclockwise movement. The metarotors sediment at the bottom of the sample cell but they are free to move laterally in response to the optical gradient force generated by the incident laser beam, resulting in stable rotation with the metarotors being slowly pulled toward the laser focal point for incident intensities *I*_0_ > ∼ 10 μW/μm^2^. Lower intensities typically did not generate consistent movement, likely due to friction against the bottom of the sample cell.

**Figure 3:**
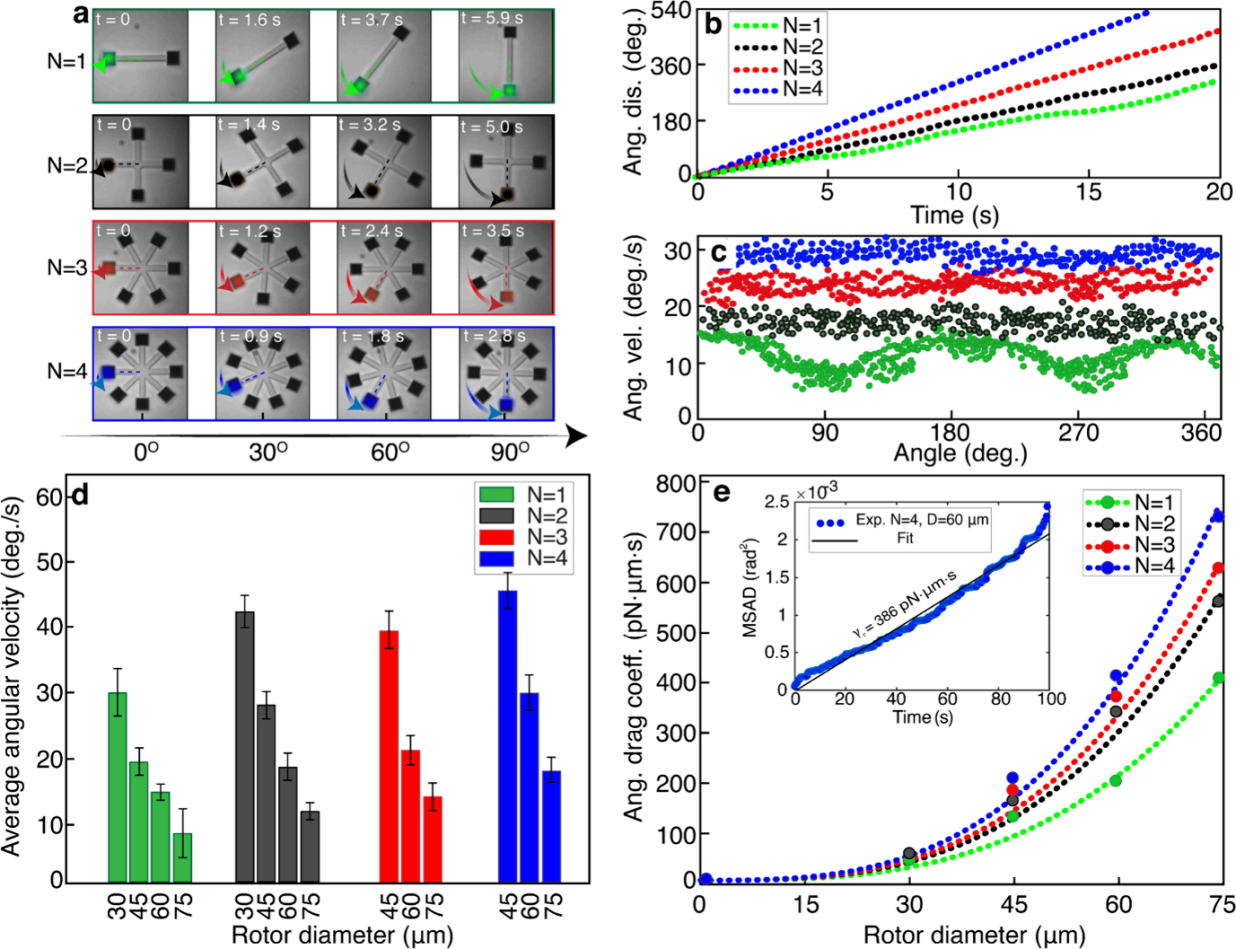
Experimental rotation data (a). Snapshots of rotating metarotors with *N* = 1, 2, 3, and 4 bars and diameters *D* = 60 μm. The rotors are equipped with square metagratings, each with *S* = 10 diffractive unit cells oriented to generate counterclockwise movement. (b) Angular displacement versus time for *N* = 1–4, *D* = 60 μm, and *S* = 10. (c) Angular velocity versus rotation angle for *N* = 1, 2, 3 and 4, *D* = 60 mm, and *S* = 10. (d) Average angular velocity for *N* = 1, 2, 3 and 4, *D* = 30–75 μm, and *S* = 10. The error bars indicate the spread in values between different samples. (e) Rotational drag coefficients γ_r_ obtained by dividing the calculated average torque from Figure 2d by the measured average angular velocities in (d). The dashed lines show fits to γ_r_ ∝ *D*^3^. The inset shows the Brownian mean square angular displacement (MSAD) versus time for a *N* = 4, *D* = 60, *S* = 10 metarotor in the absence of any driving torque. The metarotors were all excited by a linearly polarized Gaussian beam with a peak intensity *I*_0_ = 12 μW/μm^2^ and beam-waist radius *w*_0_ = 67.5 μm.

Figure 3b and c show the angular displacement φ(*t*) and the corresponding angular velocity versus rotation angle φ̇(φ), respectively, for the metarotors shown in Figure 3a (additional data for *D* = 30, 45, and 75 μm in Figure S5). Consistent with the simulation results in Figure 2, the single barrotor exhibits clear angular dependence with the highest velocity at φ = ∼ 0 and ∼180 deg., corresponding to the metagrating lines being aligned perpendicular to the incident polarization (p-polarized diffraction), while metarotors with more bars exhibit almost angular independent rotation speeds that increase with the number of bars *N*.

Figure 3d summarizes the measured average angular velocites ⟨φ̇⟩ for all fabricated *S* = 10 metarotors (additional data for *S* = 5 and *S* = 15 unit cells in Figure S6). The data clearly shows that ⟨φ̇⟩ *decreases* with increasing diameter for all *N* despite the *increase* in torque expected from Figure 2d. To understand this apperent disrepancy, one needs to take friction into account. For the dimensions and speeds involved, the Reynolds number *Re* ≪ 1 and inertia plays essentially no role, meaning that rotational drag exactly balances applied torque. Thus, τ(*t*) = γ_r_ φ̇(*t*), where γ_r_ is the rotational drag coefficient. Since γ_r_ can be expected to increase with the cube of the diameter for the essentially flat metarotors, while torque only increases linearly with the increase in lever arm length, the rotation speed should in fact decrease. To quantify this effect, we estimated γ_r_ by dividing the calculated average torques from Figure 2d by the measured average angular velocities from Figure 3d. As shown in Figure 3 e), the resulting data points can be fitted well with a cubic dependence γ_r_ ∝ *D*^3^, expected for a thin rotor, with a proportionality constant that increases gradually with *N*. As an independent consistency check, we show in the inset a measurement of γ_r_ for a *N* = 4, *D* = 60 μm rotor based on recording its in-plane mean square angular displacement (MSAD, see Methods and Figure S7), which is expected to follow ⟨φ^2^ (*t*)⟩ = 2*k*_*B*_*Tt*/γ_r_, where *k*_*B*_ is the Boltzmann constant and *T* is absolute temperature. In the absence of laser illumination, we set *T* = *T*_0_ = 297 K, which yields γ_r_ ≈ 390 pN·μm·s in good agreement with the value obtained from the fitted data (γ_r_ = 440 ± 50 pN·μm·s).

The rotational friction coefficient is directly proportional to the viscosity η of the surrounding medium, which in the case of water decreases rapidly with increasing temperature. This makes it possible to estimate the photothermal heating, Δ*T*, of a metarotor, induced by the incident laser beam, by recording its average rotational frequency versus applied intensity *I*_0_ and using ⟨φ̇⟩ ∝ τ/γ_r_ ∝ *I*_0_/(η(*T*_0_ + Δ*T*(*I*_0_)). Data for *D* = 60 μm metarotors (Figure S8) show a supralinear trend in ⟨*φ̇(I*_0_)⟩ for *I*_0_ > ∼ 50 μW/μm^2^, indicating heating, while thermal simulations (Figure S9) indicate Δ*T* ≈ 30 K close to the metagratings for the highest intensity, *I*_0_ = 120 μW/μm^2^, used in these experiments. Thus, we expect that photothermal heating is only of the order of a few degrees in the experiments based on a ten times lower intensity shown in Figure 3.

The potential application of a metarotor crucially depends on its mechanical capabilities, specifically its ability to move the surrounding fluid and nearby objects. To assess this potential, we performed finite element fluid dynamics simulations (see Methods) for *D* = 60 μm, *N* = 4, *S* = 10 metarotors at room temperature in water, as summarized in Figure 4. To perform these simulations accurately, it is necessary to estimate the distance *d* between the metarotor and the bottom surface of the measurement chamber, which imposes a no-slip boundary condition on the induced flows and thus tends to increase rotational drag compared to a rotor in bulk water. We thus fixed the metarotor angular speed to φ̇ = 50 deg·s^–1^ and then integrated the fluid viscous stress moment over its surface to obtain the fluid torque *τ*_*fluid*_ on the rotor, which yields the rotational friction coefficient as γ_r_(*d*) = *τ*_*fluid*_ (*d*)/φ̇ for comparison to experiments. In Figure 4a, we show γ_r_(*d*) normalized to it value for *d =* 25 μm, γ_r_ = 183 pN·μm·s, representing a metarotor unaffected by the boundary. A comparison to the measured γ_r_ values above then yields that the distance from the bottom surface is approximately 282 nm. Figure 4b provides the corresponding variation in fluid velocity magnitude along the radial and normal directions while Figure 4c shows a detailed velocity profile map for this distance. The data shows that the fluid velocity decays rapidly with distance. In particular, the decay length in the radial direction is only a small fraction of the metarotor radius. This is due to two primary factors: the thin profile of the metarotor and the presence of the no-slip boundary at the chamber’s bottom.

**Figure 4:**
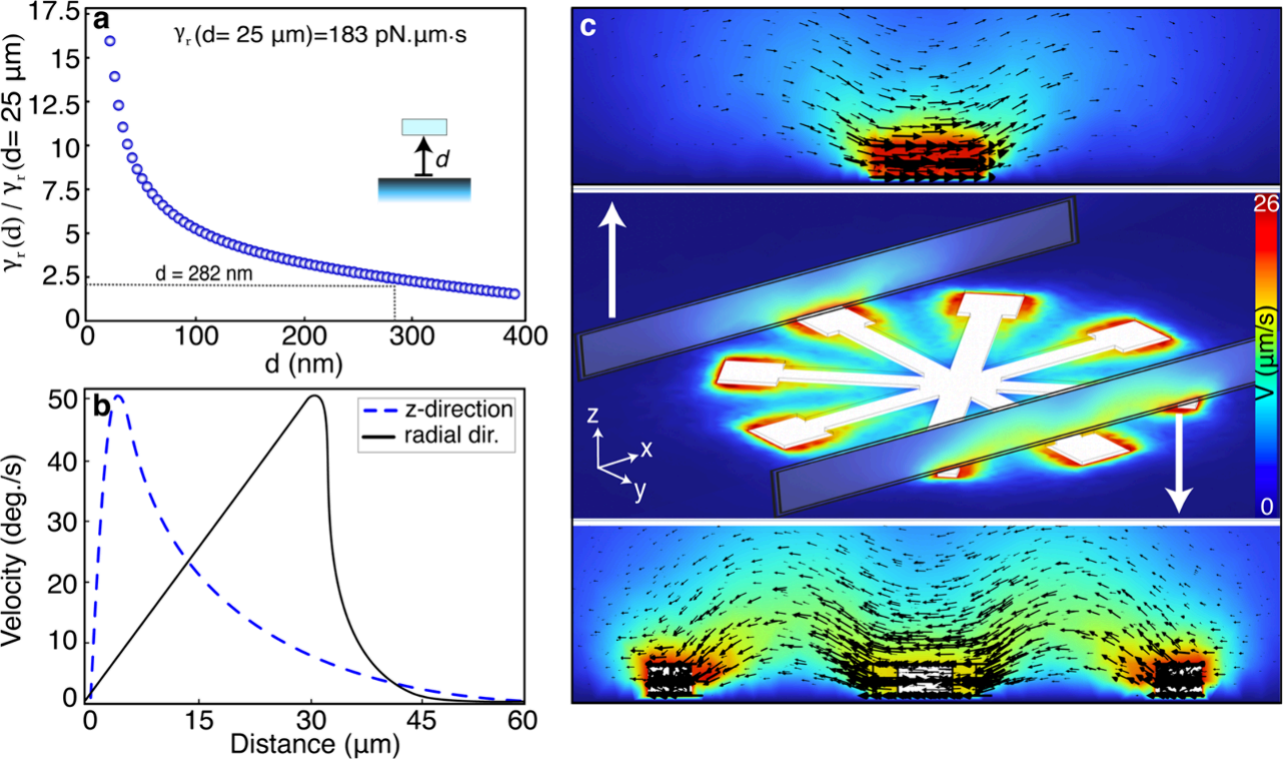
Simulated flows around a rotating metarotor. (a) Simulated rotational friction coefficient *γ*_*r*_(*d*), where *d* is the distance to the bottom no-slip boundary, of a *D* = 60 μm, *N* = 4, *S* = 10 metarotor that rotate at room-temperature in water. The data have been normalized to *γ*_*r*_ at *d* = 25 μm, representing a “free” metarotor, and the lines at *d* = 282 nm mark the measured drag from Figure 3e. (b) Simulated average fluid velocity magnitude variation along the radial direction just above the rotor (z = 1.1 μm) and in the normal direction at r = 30 μm. The metarotor rotates with an angular speed of 50 deg.s^–1^ at *d* = 282 nm. (c) Flow profiles around the metarotor. The upper and lower panels show vertical flow vector profiles at the inner and outer periphery of a metagrating, as indicated in the central panel.

The small lateral extension of the flow profile seen in Figure 4 indicates that the metarotors are poor stirrers of their liquid environment, as is expected for any rotor operating at low Reynolds number. However, as demonstrated in Figure 5, this does not imply that they are unable to rotate surrounding material. In this experiment, we introduced 7 μm diameter polystyrene (PS) beads into the measurement chamber and then recorded their movements as the *D* = 60 μm metarotor rotated at constant applied optical torque provided by the incident Gaussian beam (*I*_0_ = 25 μW/μm^2^). Because of their comparatively large volume, the PS beads are first attracted toward the metarotor by the optical gradient force, possibly amplified by weak thermal convection flows,^30^ and then begin to follow its motion. As more and more beads are pulled in, they successively form concentric rings and fill up the space between and on top of the bars, while the metagratings remains essentially uncovered (Figure 5a and Video 5). In Figure 5b, we show that the angular velocity of the metarotor decreases linearly by ∼24%, corresponding to a ∼ 32% increase in γ_r_, during the 5 min time course of the experiment. During this time, the rotor has acquired five concentric PS bead rings (∼200 particles in total). The velocities of the bead rings decrease successively outward, but with a slower rate than the simulated flow speed at the corresponding radial distance (Figure 4b), indicating that their angular movement is driven by a combination of fluid flow and mechanical friction between the beads. As a further example of the possibility of transferring torque from a metarotor to passive objects, Supplementary Video 6 shows how a *D* = 30 μm, *N* = 2 rotor can drive a passive *D* = 60 μm, *N* = 2 structure that does not contain any light deflecting metasurfaces. Finally, the results discussed thus far all refer to single isolated metarotors but there is no restriction in the number of metarotors that can be driven by a single laser beam, as long as it covers a large enough area. As an example, Supplementary Video 7 shows how several *N* = 2 rotors with different diameters rotate together within the large field of view.

**Figure 5:**
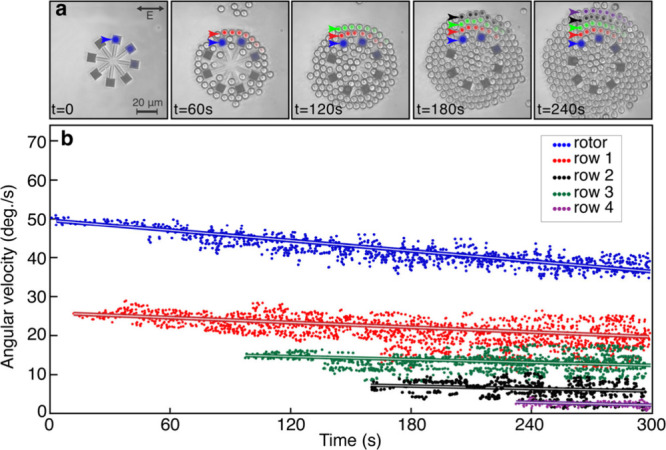
Rotation of 7 μm diameter polystyrene beads. (a) Sequential snapshots of a *D* = 60 μm, *N* = 4, *S* = 10 metarotor that is initially rotating at 50 deg/s as PS beads are pulled in by the optical gradient force and rotated by the metarotor. The linearly polarized incident beam has peak intensity *I*_0_ = 25 μW/μm^2^ and beam waist *w*_0_ = 67.5 μm. (b) Angular velocities of the metarotor (blue points) and four successive rings of PS beads (as indicated in (a)) versus time.

In summary, we have demonstrated that metarotors–dielectric structures that are optically thin but many tens of micrometers in diameter–can be operated as effective light driven rotary micromachines or microrobots when equipped with symmetrically arranged metagratings that deflect light azimuthally at a high angle. We have characterized the performance of the metarotors in an aqueous environment and shown that their rotational speed can be quantitatively explained by considering the orbital angular momentum torque created through light deflection and the rotational Stokes drag near the supporting substrate. As proof of principle application, we showed that a 60 μm diameter rotor equipped with 8 metagratings was able to rotate at least 200 beads with 7 μm diameters in solution. The size of these beads is of the same order as many biologically relevant entities, such as yeast cells and erythrocytes, indicating that the metarotors could be potentially useful in microbiology experiments and applications. The remarkable mechanical power transfer ability of the metarotors additionally suggests promising applications in microfluidics, such as mixers, pumps, and gates.

## Data Availability

Data are available from the authors upon reasonable request.

## Supporting Information Available

Methods, sample fabrication process, metagrating efficiency, optical setup, additional experimental data, and thermal simulations (PDF)
Video 1: Rotation of a single bar metarotor (MP4)
Video 2: Rotation of a double bar metarotor (MP4)
Video 3: Rotation of a triple bar metarotor (MP4)
Video 4: Rotation of a quadruple bar metarotor (MP4)
Video 5: Interaction of a rotary quadruple bar robot (MP4)
Video 6: Example of transferring an optical torque (MP4)
Video 7: Example of simultaneous rotation of metarotors (MP4)
Video 8: Rotation of a quadruple bar metarotor (MP4)
Vidoe 9: CFD simulation of a quadruple bar metarotor (MP4)
