# Supplementary material for: Harnessing Photon Recoil for Enhanced Torque on Light-Driven Metarotors
Source: Nano Lett. 2025 Mar 3;25(12):4832–7. doi: 10.1021/acs.nanolett.4c06410 (PMC11951154; doi:10.1021/acs.nanolett.4c06410)
Supplement: Supplementary file 2 — nl4c06410_si_003.pdf [file nl4c06410_si_003.pdf]

Harnessing photon recoil for enhanced torque on light-driven metarotors

Methods, Supporting Figures, and Supporting Videos

Mahdi Shanei[1], Gan Wang[2], Peter Johansson[3], Giovanni Volpe[2], and Mikael Käll[1*]

[1]Department of Physics, Chalmers University of Technology, Gothenburg Sweden
[2]Department of Physics, University of Gothenburg, Gothenburg, Sweden
[3]School of Science and Technology, Örebro University, 701 82 Örebro, Sweden
*Corresponding author: mikael.kall@chalmers.se

METHODS

Metarotor fabrication was based on the metagrating design reported in [30], with unit cell width 817 nm, ridge height 490 nm, distance between ridges 100 nm, and ridge widths 190, 130, and 90 nm. Fabrication commences on a 4-inch silicon (Si) wafer with a 390 nm layer SiO_2 layer and a 490 nm thick amorphous silicon (a-Si) layer. Ellipsometry characterization of the resulting a-Si layer gave a complex refractive index of $n_{a\text{-}Si} = 3.8 + i0.0064$. The a-Si metagratings and the SiO_2 body that constitute a metarotor are then defined using a sequence of electron beam lithography (EBL) and etching steps, as schematically illustrated in Supporting Fig S1 and scanning electron microscopy images in Fig S2. In the first series of steps, the metagratings are defined by first spin coating a positive resist mask (ARP6200.13) onto the a-Si. The resist is baked at 160°C for 5 min, exposed at 310 $\mu C/cm^2$ (10 nA), and developed for 90 sec. To facilitate further structural definition, a hard mask of 3 nm Cr and 55 nm Ni is deposited on the resist. The metagrating patterns are then transferred to the a-Si layer through Cl_2 reactive ion etching step (50 SCCM Cl_2 at 10 mTorr and 50/100 W of power on the inductively coupled plasma (ICW) and the forward power (FW) electrodes, respectively. The remaining hard mask is then removed through wet etching with Microposit Remover 1165 at 80 ˚C. In a second series of steps, the body of the metarotors are defined. The metagratings are first covered by a 610 nm SiO_2 layer using plasma-enhanced chemical vapor deposition (210 SCCM SiH_4, 50 SCCM N_2O at 350 mTorr). A second EBL step is performed to define the body structures, following the same recipe as used for the metagrating fabrication. After this, a combination of hard mask evaporation and lift-off is executed. The PECVD and thermally deposited SiO_2 layers are etched using CHF_3 (12 SCCM CHF_3 and 17 SCCM Ar, 5 mtorr, 580/50 W ICP/FW). The residual hard mask is subsequently removed through wet etching. In the final fabrication step, the metarotors are released from the Si wafer through isotropic etching in SF_6 (50 SCCM SF_6 at 20 mTorr and 350/15 W ICP/FW) for ~17 min. and then dispersed in deionized water.

Electrodynamics simulations were conducted using finite element simulations in Comsol Multiphysics using metagrating parameters as above and in-plane periodic boundary conditions. The a-Si (n = 3.8) structure was enclosed by a 1 μm thick SiO_2 (n = 1.45) slab submerged in water (n = 1.33). The normally incident source field with wavelength λ_0 = 1064 nm was linearly polarized with an angle φ with respect to the diffraction plane. The transmission and reflection efficiencies of the six allowed diffraction orders were recorded as a function of φ, as displayed in Supporting Fig. S3.

Optical rotation experiments were performed with the metarotors submerged in a ~4 μl sample cell made from two microscope slides separated by a 120 μm spacer and filled with de-ionized water. The sample cell was mounted on an inverted microscope (Nikon Ti) and the metarotors were imaged from below using a dry objective (Nikon 40x, NA = 0.95). The driving laser beam (λ_0 = 1064 nm, Cobolt Rumba 2W), incident from above, was focused to a Gaussian spot with beam-waist radius $w_0 \approx 67.5$ μm. A schematic of the setup is shown in Supporting Fig. S4. Normal video tracking of metarotor movements was performed at 34 frames per second using standard tracking software. The Brownian angular displacement measurement was performed at an increased rate of 250 frames per second by zooming in on a single metagrating, tracking the positions (x,y) of two adjacent corners, A and B, and calculating the angle relative to the laboratory frame as $\varphi = \arctan\,[(y_B - y_A)/(x_B - x_A)]$.

Fluid dynamics simulations were performed in COMSOL Multiphysics using the Rotating Machinery, Laminar Flow interface to solve Navier-Stokes equations under laminar flow conditions. We simulated the behavior of a 60 μm diameter metarotor with four arms and 10x10 μm^2 metagratings in room-temperature water (Fig. 4a). The rotor is centered at the axis of a cylindrical calculation domain (diameter 300 μm, height 50 μm) at a distance d from its bottom. The bottom and top surfaces of the calculation domain, as well as the metarotor interface, obey no-slip boundary conditions while the domain sidewall boundaries permit fluid inflow and outflow. We utilized two distinct mesh domains: a moving domain with max(min) mesh size 1(0.1) μm, containing the metarotor, and a stationary domain with max(min) mesh-size of 5(1) μm, encompassing the rest of the model. A flow continuity condition was applied to the boundary between these domains.

Thermal simulations of the temperature distribution around a 10x10 μm^2 metagrating was performed using COMSOL Multiphysics. We first calculated the absorption cross-section σ_{abs} at λ_0 = 1064 nm for a metagrating unit cell with dimensions 0.817x10x1 μm^3 and containing the a-Si grating ridges ($n_{\text{a-Si}}$ = 3.8 + i0.0064) embedded in SiO_2. The corresponding unit cell heat source density, $Q=\sigma_{abs}I/V$, where I is the incident intensity and V is the unit cell volume, then defines the heat generated within a complete metasurface containing several unit cells. The resulting temperature distribution in the vicinity of the metagrating was obtained by considering heat transfer through conduction and convection in a 1x1x0.1 mm^3 water volume with room-temperature boundaries.

SUPPORTING FIGURES

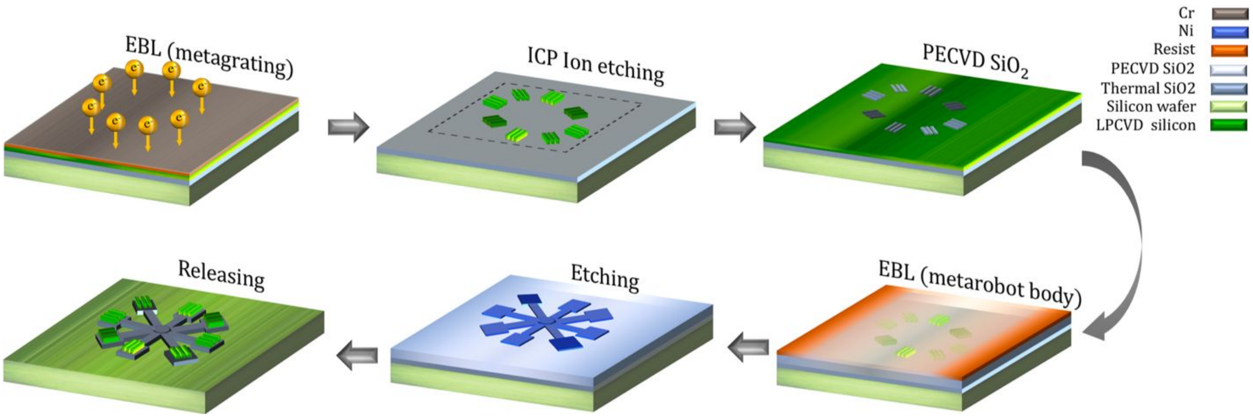

Supporting Figure S1. Fabrication process. Schematic illustrations of some of the key steps involved in making metarotors. The top row illustrates fabrication of the metagratings, which involves patterning a resist using electron beam lithography (EBL), transferring the pattern to amorphous Si through inductively coupled plasma (ICP) etching, and coating the metagratings with SiO_2 to protect them during the final release step. The bottom row illustrates the fabrication of the metarotor body, which includes a second-layer EBL exposure and etching. The metarotors are finally detached from the substrate through isotropic etching and released into water.

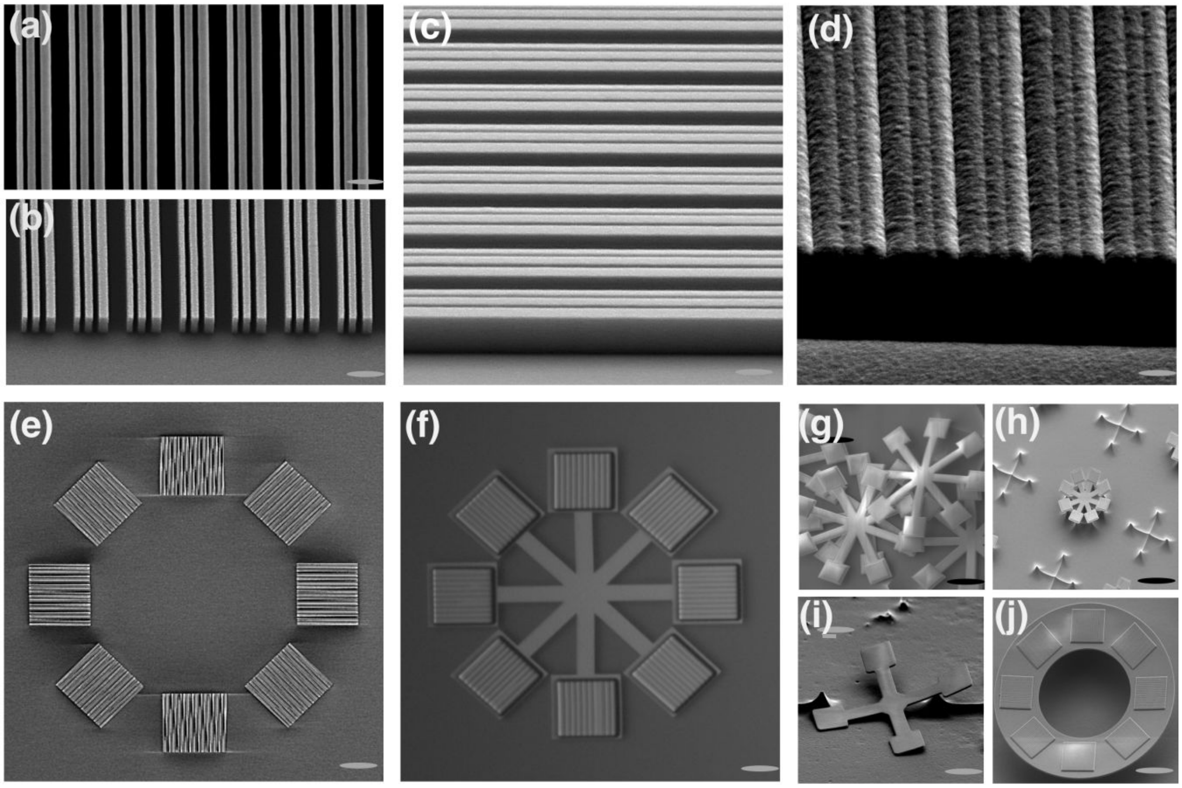

Supporting Figure S2. Scanning electron microscopy images from various steps in the fabrication process. (a) Top, (b) tilted, and (c) side views of aSi metagratings after first layer exposure and etching (scale bar 500 nm). (d) Image of a metagrating covered with protective SiO_2 (scale bar 500 nm). (e,f) Top views of a quadruple metarotor containing eight square metagratings (e) before and (f) after defining the metarotor body (scale bar 7 μm). (g-j) Images of different structures investigated during the fine-tuning of the fabrication procedure: (g) and (i) show body structures (scale bar 10 μm), (h) show metarotors ready for release (scale bar 40 μm), and (j) shows a quadruple metarotor with an alternative body design (scale bar 10 μm).

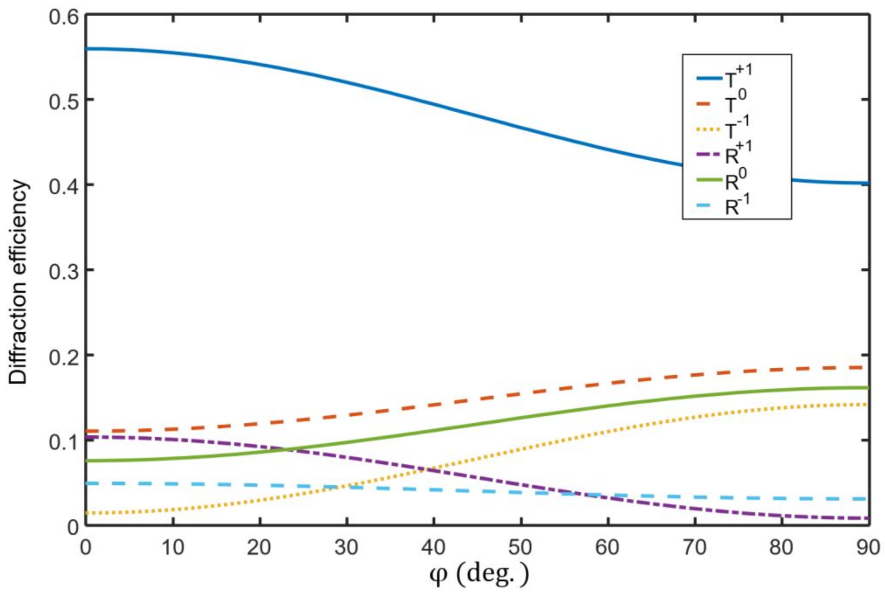

Supporting Figure S3. Calculated metagrating diffraction efficiencies. The figure shows power transmission (T) and reflection (R) diffraction efficiencies for the three allowed orders (+1, 0, -1) versus angle φ between the plane of diffraction and the plane of linear polarization obtained from electrodynamic finite element simulations using the same metagrating parameters as in the fabricated samples, normal incidence, and in-plane periodic boundary conditions. The aSi metagrating is positioned symmetrically in a 1 μm thick slab of SiO_2 in a water environment. Angles $\varphi = 0$ and $\varphi = 90$ deg. correspond to p-polarized and s-polarized diffraction, respectively.

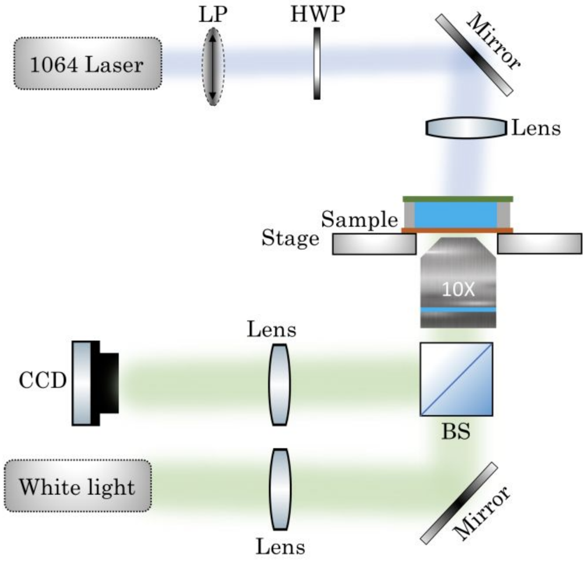

Supporting Figure S4. Experimental setup for metarotor characterizations. The metarotors are situated in a thin liquid cell mounted on an inverted microscope and viewed in reflection using a microscope objective and white light illumination from below. Laser light with wavelength λ_0 = 1064 nm is adjusted in polarization using a half-wave plate (HWP) and focused on the sample plane using a lens with focal length 50 mm, resulting in a Gaussian intensity distribution with beam-waist radius $w_0 \approx$ 67.5 μm.

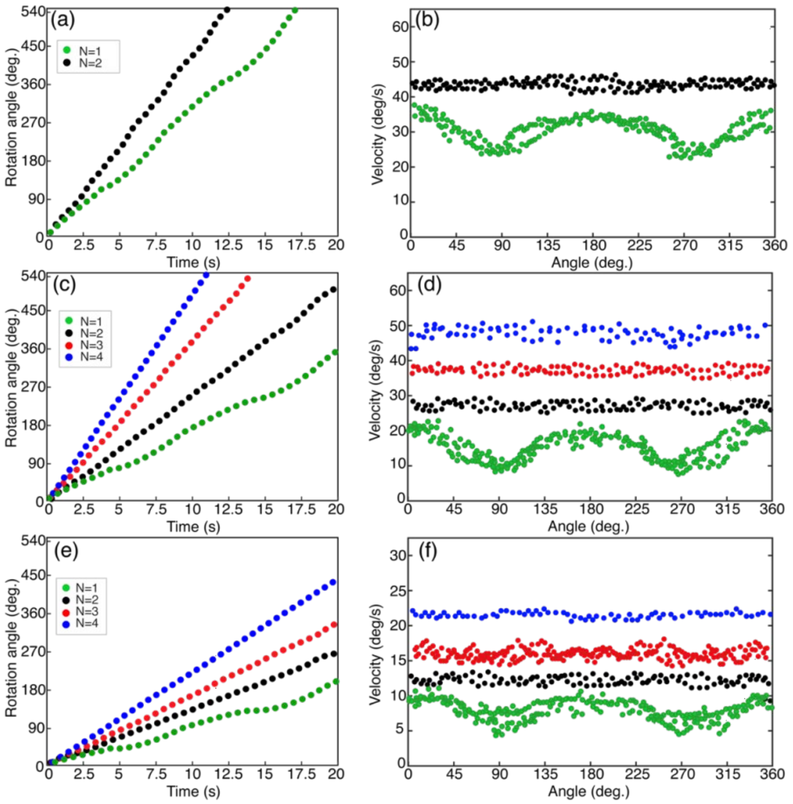

Supporting Figure S5. Metarotor rotation dynamics. Angular displacement versus time (left column) and corresponding angular velocity versus rotation angle φ (right column) for metarotors with different number of SiO_2 bars N and diameters (a-b) D = 30 µm, (c-d) D = 45 µm, and (e-f) D = 75 mm. The illumination conditions are the same as in main Fig. 3 b-c).

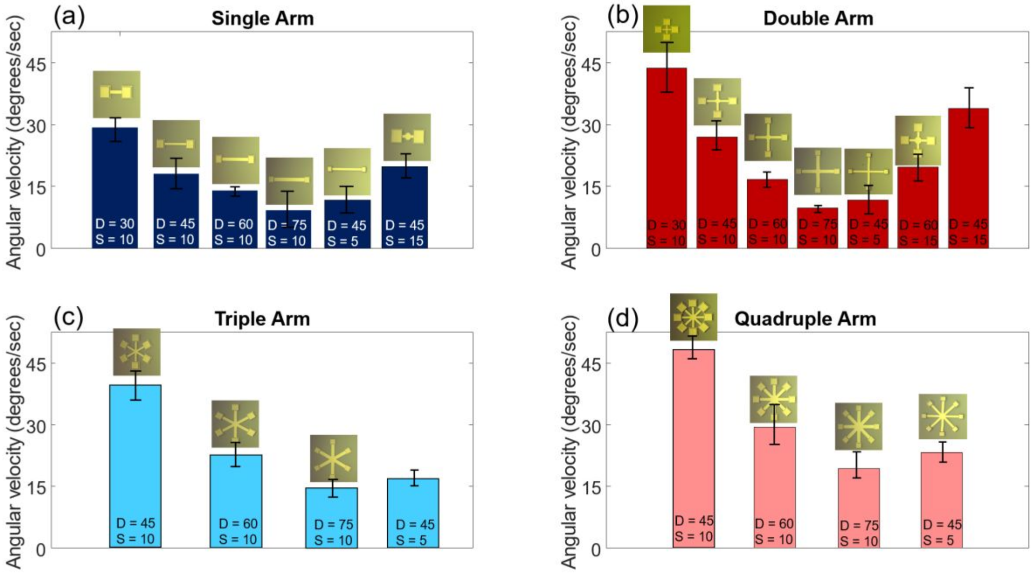

Supporting Figure S6. Measured average angular velocity for full metarotor dataset. Average angular velocities for (a) single-bar (N = 1), (b) double-bar (N = 2), (c) triple-bar (N = 3), and (d) quadruple-bar (N = 4) metarotors with diameters D [µm] and equipped with metagratings containing S unit cells, as indicated in the figures, together with corresponding scanning electron micrographs. The illumination conditions are the same as in main Fig. 3 d).

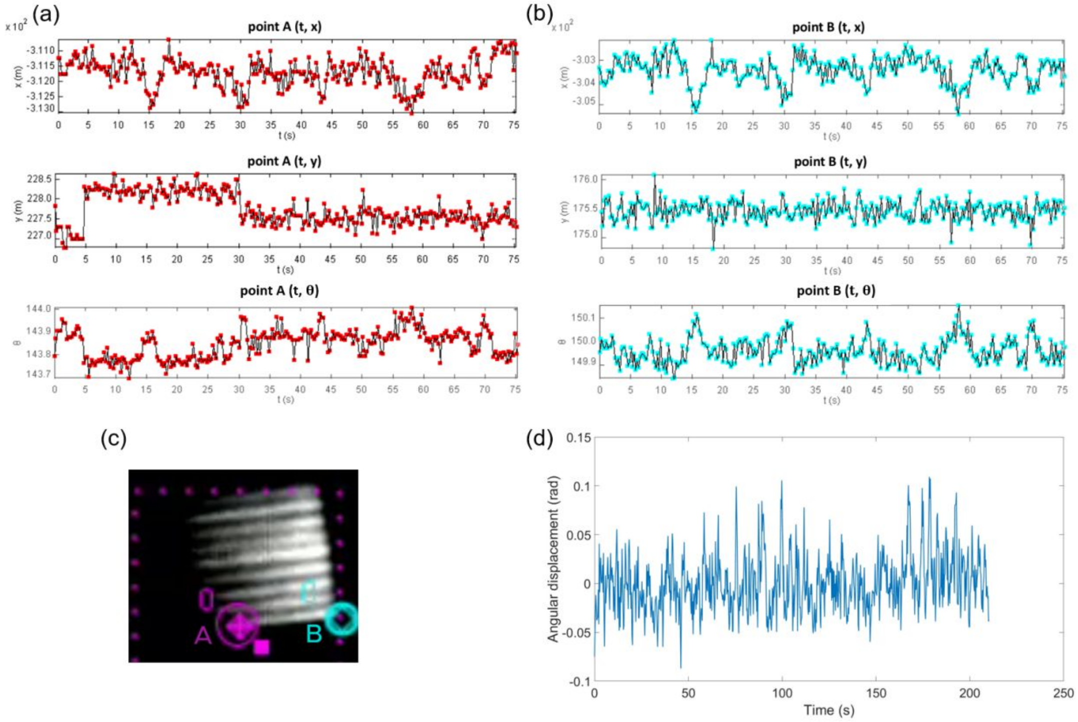

Supporting Figure S7. Estimate of rotation drag coefficient through measurement of Brownian diffusion. (a) and (b) A part of tracking of positions (x, y) of two neighboring corners, A and B, on one of the metagratings belonging to a $N = 4$, $S = 10$, $D = 60$ μm metarotor. (c) video grab showing tracking points A and B. (d) Extracted angular displacement $\varphi(t)$ calculated from the positions of A and B. The measurements were performed in white light illumination with the driving laser turned off.

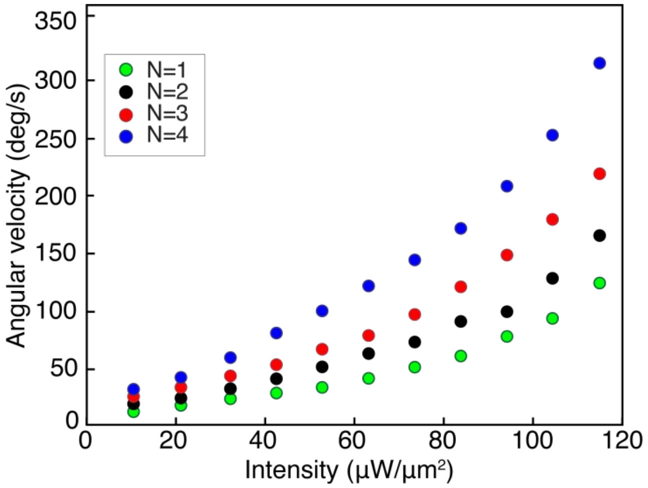

Supporting Figure S8. Rotation speed versus incident intensity. Average angular velocity of $D = 60$ μm metarotors with $N = 1$-4 SiO_2 bars and $S = 10$ metagratings as a function of peak intensity I_0 of the incident Gaussian laser beam. Note the supralinear trend for higher intensities.

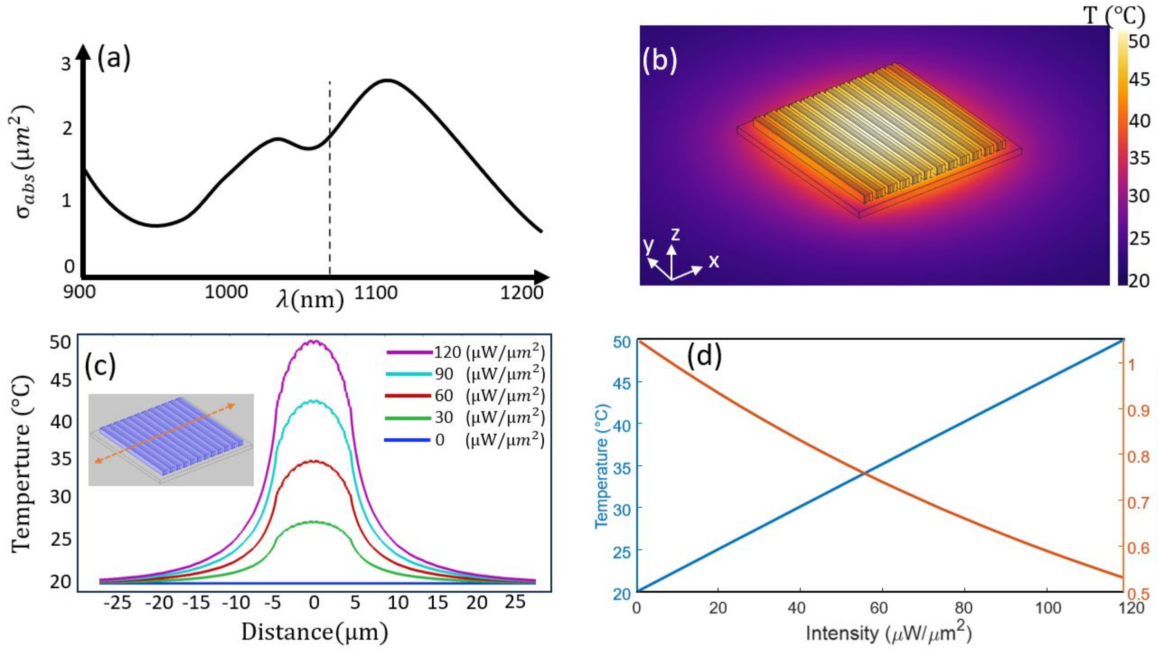

Supporting Figure S9. Photothermal heating of a metagrating. (a) Absorption cross-section spectrum for the 0.817 μm × 10 μm unit cell of a 10x10 μm^2 metagrating from electrodynamic finite element simulations based on a complex aSi refractive index of n_{aSi} = 3.8 + i0.0064. The incident field is polarized parallel to the grating lines (s-polarization). The corresponding cross-section for p-polarization was $\sigma_{abs} = 1.66\, \mu m^2$ at λ_0 = 1064 nm. (b) Thermal finite element simulation of the temperature distribution around a 10x10 μm^2 metagrating based on the calculated absorption cross-section for s-polarization at λ_0 = 1064 nm (see Methods). The driving light intensity corresponds to the case of a 60 μm diameter metarotor in a Gaussian beam with peak intensity I_0 = 120 μW/μm² and w_0 = 67.5 μm. (c) Simulated thermal distribution along a line on the top of the metagrating, as indicated by the inset, for different peak intensities I_0. (d) Maximum temperature around the metagratings as a function of incident peak intensity and the corresponding dynamic viscosity of water.

SUPPORTING VIDEOS

Supporting Video 1: Rotation of a single-bar metarotor with diameter 60 μm by a horizontally polarized incident beam with $I \approx 75\ \mu W \cdot \mu m^{-2}$.

Supporting Video 2: Rotation of a double-bar metarotor with diameter of 60 μm by a horizontally polarized incident beam with $I \approx 75\ \mu W \cdot \mu m^{-2}$.

Supporting Video 3: Rotation of a triple-bar metarotor with diameter 60 μm by a horizontally polarized incident with $I \approx 75\ \mu W \cdot \mu m^{-2}$.

Supporting Video 4: Rotation of a quadruple-bar metarotor with diameter 60 μm by a horizontally polarized incident beam with $I \approx 75\ \mu W \cdot \mu m^{-2}$.

Supporting Video 5: Interaction between a quadruple-bar metarotor with diameter 60 μm and polystyrene beads with diameter 7 μm. Note the doubling of the display speed at $t \approx 40$ s.

Supporting Video 6: Example of transferring optical torque from a 30 μm diameter metarotor to a passive 60 μm structure without metagratings.

Supporting Video 7: Example of simultaneous rotation of two metarotors, with diameters 100 μm and 50 μm, within the field of view.

Supporting Video 8: Rotation of a quadruple-bar metarotor with diameter 60 μm for various incident intensities.

Supporting Video 9: Computational Fluid Dynamics simulation of a quadruple-bar metarotor with diameter 60 μm. The insets show the flow at the indicated cross-section.

All videos play in real time, unless indicated, and show metarotors with metagrating sizes of $10\ \mu m \times 10\ \mu m$.
